# Urinary metabolic profiles in early pregnancy are associated with preterm birth and fetal growth restriction in the Rhea mother–child cohort study

**DOI:** 10.1186/1741-7015-12-110

**Published:** 2014-07-11

**Authors:** Léa Maitre, Eleni Fthenou, Toby Athersuch, Muireann Coen, Mireille B Toledano, Elaine Holmes, Manolis Kogevinas, Leda Chatzi, Hector C Keun

**Affiliations:** 1 Computational and Systems Medicine, Department of Surgery and Cancer, Faculty of Medicine, Imperial College London, London SW7 2AZ, UK; 2 Department of Social Medicine, Faculty of Medicine, University of Crete, PO Box 2208, 71003 Heraklion, Greece; 3 Centre for Research in Environmental Epidemiology (CREAL), Barcelona, Spain; 4 MRC-PHE Centre for Environment and Health, Imperial College London, London W2 1PG, UK; 5 Department of Epidemiology and Biostatistics, School of Public Health, Faculty of Medicine, Imperial College London, London W2 1PG, UK; 6 National School of Public Health, Alexandras Avenue 196, 115 21 Athens, Greece; 7 IMIM (Hospital del Mar Research Institute), Barcelona, Spain; 8 CIBER Epidemiologia y Salud Pública (CIBERESP), Barcelona, Spain

**Keywords:** Fetal growth restriction (FGR), Intrauterine growth restriction (IUGR), Small for gestational age (SGA), Preterm birth (PB), NMR, Metabonomics, Metabolomics, *In utero* environment, Exposome

## Abstract

**Background:**

Preterm birth (PB) and fetal growth restriction (FGR) convey the highest risk of perinatal mortality and morbidity, as well as increasing the chance of developing chronic disease in later life. Identifying early in pregnancy the unfavourable maternal conditions that can predict poor birth outcomes could help their prevention and management. Here we used an exploratory metabolic profiling approach (metabolomics) to investigate the association between birth outcomes and metabolites in maternal urine collected early in pregnancy as part of the prospective mother–child cohort Rhea study. Metabolomic techniques can simultaneously capture information about genotype and its interaction with the accumulated exposures experienced by an individual from their diet, environment, physical activity or disease (the exposome). As metabolic syndrome has previously been shown to be associated with PB in this cohort, we sought to gain further insight into PB-linked metabolic phenotypes and to define new predictive biomarkers.

**Methods:**

Our study was a case–control study nested within the Rhea cohort. Major metabolites (n = 34) in maternal urine samples collected at the end of the first trimester (n = 438) were measured using proton nuclear magnetic resonance spectroscopy. In addition to PB, we used FGR in weight and small for gestational age as study endpoints.

**Results:**

We observed significant associations between FGR and decreased urinary acetate (interquartile odds ratio (IOR) = 0.18 CI 0.04 to 0.60), formate (IOR = 0.24 CI 0.07 to 0.71), tyrosine (IOR = 0.27 CI 0.08 to 0.81) and trimethylamine (IOR = 0.14 CI 0.04 to 0.40) adjusting for maternal education, maternal age, parity, and smoking during pregnancy. These metabolites were inversely correlated with blood insulin. Women with clinically induced PB (IPB) had a significant increase in a glycoprotein *N-*acetyl resonance (IOR = 5.84 CI 1.44 to 39.50). This resonance was positively correlated with body mass index, and stratified analysis confirmed that *N-*acetyl glycoprotein and IPB were significantly associated in overweight and obese women only. Spontaneous PB cases were associated with elevated urinary lysine (IOR = 2.79 CI 1.20 to 6.98) and lower formate levels (IOR = 0.42 CI 0.19 to 0.94).

**Conclusions:**

Urinary metabolites measured at the end of the first trimester are associated with increased risk of negative birth outcomes, and provide novel information about the possible mechanisms leading to adverse pregnancies in the Rhea cohort. This study emphasizes the potential of metabolic profiling of urine as a means to identify novel non-invasive biomarkers of PB and FGR risk.

## Background

Fetal growth restriction (FGR) and preterm birth (PB) are the main predictors of perinatal morbidity and mortality [1,2]. These birth outcomes are associated with growth failure and accelerated weight gain during childhood. As a consequence, adverse child health and predisposition to metabolic and cardiovascular disorders can appear later in life [3,4]. Over the past 10 years, PB has increased by 19.4% in developed regions with the USA, accounting for 42% of these preterm births in 2010 [5]. PB can either be medically induced on the basis of maternal or fetal indications, or spontaneously induced. Spontaneous PB (SPB) occurs at different prevalences in different ethnic groups and is believed to be associated with intrauterine infection (25 to 40% of cases), uterine overdistension due to multiple gestations, PB history, or shortened cervix [6]. Medically induced PB (IPB), which depends upon the decision of the clinician, often reflect underlying conditions involved with obesity such as pregnancy-induced hypertension or pre-gestational diabetes. FGR, which represents pathological inhibition of fetal growth and failure of the fetus to attain its growth potential, can be due to fetal genetic abnormalities or congenital infections (for example, toxoplasmosis, malaria, rubella). However, the vast majority of FGR cases are the result of extrinsic factors comprising maternal and placental conditions, such as placental ischemia and uteroplacental deficiency [7]. In the developed world, FGR is prevalent in women with hypertensive disorders, exposure to toxins (in particular cigarette smoke) and poor nutritional status [8-10].

A recent report evocated a sharp increase in late PB in Greece for the past 20 years, in a similar fashion to what has been noted in other middle or high income countries, potentially associated with increased maternal age and a change in obstetric interventions [11]. Other factors were reported in several studies with associations between pre-pregnancy metabolic disease, such as obesity [12-14], chronic hypertension [15,16], dyslipidaemia and inflammation in early pregnancy [17] and high risk of PB. To better understand the underlying mechanisms that give rise to PB and FGR, the present study used data from the Rhea cohort, a large population-based mother–child cohort initiated in Crete in 2007 [18]. In this cohort, women with metabolic syndrome early in pregnancy were at high risk for PB (relative risk (RR) = 2.93, 95% CI 1.53 to 5.58), with the highest risk observed for IPB (RR = 5.13, 95% CI 1.97 to 13.38). Because routine prenatal care fails to identify a large proportion of women at risk, a better understanding of birth outcomes is crucial to improve their prediction and prevention. The application of metabolic profiling (metabolomics/metabonomics) to pregnancy research has emerged mainly as a non-targeted approach to explore potential biomarkers of reproductive outcomes and identify underlying biological mechanisms [19-21]. It has been suggested that the use of molecular biomarkers in combination with fetal monitoring and other maternal characteristics may be of clinical benefit [22,23]. To achieve this, new, large, prospective cohort studies are needed in which biospecimen characterisation is coupled with detailed analysis of maternal physiology, lifestyle and medical history. Although other studies using metabolomics to investigate PB and FGR risk factors have been reported, to our knowledge this current study represents the largest human investigation (n = 438) to date in which urinary metabolomics has been used to identify metabolite predictors early in pregnancy (11-13 weeks).

## Material and methods

### Ethics statement

The study was conducted according to the guidelines laid down in the Declaration of Helsinki, and all procedures involving human subjects were approved by the ethical committee of the University Hospital in Heraklion, Crete, Greece. Written informed consent was obtained from all women participating in the study.

### The mother-child cohort in Crete, Rhea study

The Rhea project is a mother–child study, prospectively examining a population-based cohort of pregnant women and their children in the prefecture of Heraklion, Crete, Greece [18]. Female residents (native Greeks and immigrants) who had become pregnant during the 12-month period starting in February 2007 were contacted at the four maternity clinics (two public and two private) in Heraklion, and asked to participate in the study. Study enrolment and urinary collection were made at the end of the first trimester, at the time of the first major ultrasound examination (mean ± SD 11.96 ± 1.49 weeks). Questionnaires on health behaviours, pregnancy history, lifestyle characteristics, and dietary habits during pregnancy were administered by trained interviewers at enrolment, during the third trimester, and at delivery.

During this study period 1,317 women were followed up until delivery. Women with incomplete diagnostic information, multiple pregnancies, diagnosed pre-eclampsia (a condition associated with PB), spontaneous or induced abortion, or who gave birth to stillborn infants were not included in the study [18]. Our metabolomics study was designed as a case–control study nested within the Rhea cohort. Mothers giving birth preterm and for whom early pregnancy urine samples were available, were matched with controls (in a ratio of approximately 1:3) based on age (±2 years), country of origin and parity (n = 464). From these urine specimens, proton nuclear magnetic resonance (^1^H-NMR) spectra were acquired, of which 26 spectra were excluded (because of high dilution or high excretion of drug metabolites), leaving 438 spectra available for modelling the metabolite profile with respect to birth outcome.

### Definition of the outcomes

PB, the primary outcome of interest, is defined as premature delivery at less than 37 weeks of gestation [24]. The gestational age was estimated as the period between the most recent menstruation and the delivery. When the date did not match the ultrasound measurement estimation by 7 days or more, the gestational age was corrected using its relationship to the crown–rump length [18]. Of the PBs, some were classified as spontaneous deliveries (SPBs; n = 88) when the birth was vaginal or when the labour was not documented as having been induced. Any PBs requiring either an induction of labour or pre-labour caesarean, or both, were defined as medically induced deliveries (IPB; n = 26) [25]. In addition, neonates were classified as FGR in weight if their birth weight fell below the 10th percentile of their predicted birth-weight distribution, adjusted for genetic growth potential. This customised estimation of growth impairment allows for better detection of those neonates who fail to reach their genetic growth potential or their constitutional potential because of maternal, fetal, placental or external factors, and excludes constitutionally small babies [26].

A multivariable fractional polynomial linear regression model was used to predict birth weight, allowing polynomial terms for continuous variables in the linear regression models. The final model included as covariates the gestational age, infant gender, maternal and paternal height, pre-pregnancy maternal weight, and interaction of gestational age with maternal weight. Gestational age and type of PB were known for 438 women, whereas FGR data were available for only 401 women because a number values necessary to define the outcome were missing.

### Metabolic syndrome variables

Data on plasma triglycerides, total cholesterol, high density lipoprotein cholesterol (HDL-C) and low density lipoprotein cholesterol (LDL-C) of 227 fasting pregnant women at the first prenatal visit were available [18]. The insulin concentrations were measured for 369 women, and the diastolic and systolic blood pressures (BPs) were available for 338 participants. The body mass index (BMI) calculated on reported weight before pregnancy and height, measured at the first prenatal visit, was used to classify women as underweight (BMI <18.5 kg/m), normal weight (BMI >18.5 to <25 kg/m), overweight (BMI 25 to 30 kg/m) or obese (BMI >30 kg/m), according to the standard international classification.

### ^1^H NMR spectroscopic analysis of urine

#### Sample handling and preparation

Urine samples were stored at −80 °C until analysis. An aliquot of 400 μL of urine was added to 200 μL phosphate buffer solution (0.2 M Na_2_HPO_4_/NaH_2_PO_4_, pH 7.4) to minimise variations in chemical shift values in the acquired ^1^H NMR spectra due to minor pH differences. This buffer contained 1 mM sodium 3-trimethylsilyl-(^2^H_4_)-1-propionate (TSP) in 20% D_2_O and 3 mM of the bacteriostatic agent sodium azide (NaN_3_). TSP is a chemical shift reference (δ = 0.00) and D_2_O provided a field-frequency lock. The buffered urine sample was then centrifuged at 16,000 × *g* for 5 minutes to remove any debris, and 550 μL of the resulting supernatant was pipetted into standard 5 mm NMR tubes [27].

#### ^1^H NMR experiments and data processing

^1^H NMR spectra of the urine samples were acquired using a Bruker Avance 600 spectrometer (Bruker Biospin, Rheinstetten, Germany) operating at 600.13 MHz. The ^1^H NMR spectra of the urine samples were acquired using a standard one-dimensional pulse sequence with water pre-saturation (recycle delay-90°-*t*_1_-90°-*t*_m_-90°-acquisition; XWIN--NMR 3.5) during both the recycle delay (2 seconds) and mixing time (t_m_, 100 milliseconds). The 90° pulse length was adjusted to approximately 10 μs and *t*_1_ was set to 3 microseconds. For each sample, 128 free induction decays (FIDs) were collected into 32 K data points using a spectral width of 12,000 Hz. The FIDs were multiplied by an exponential weighting function corresponding to a line broadening of 0.3 Hz prior to Fourier transformation [27].

All NMR spectra (spectral region δ 10 to 0.5) were imported into MATLAB 7.3.1 (MathWorks), and were referenced and corrected for phase and baseline distortion using an in-house script (developed by Drs Rachel Cavill, Hector Keun and Tim Ebbels, Imperial College, London, UK). The spectral region δ 4.0 to 5.4, containing residual water and urea resonances, were removed prior to median fold change normalisation [28]. Integrals of well-resolved peaks were calculated. Certain metabolites were quantified using the Profiler and Library Manager modules in Chenomx NMRSuite 5.11 (Chenomx Inc, Edmonton, Canada), when overlapping signals were present in the integration window or when there were metabolites with a low signal-to-noise ratio (specifically creatine, creatinine, tyrosine, dimethylamine (DMA) and 1-methylnicotinamide). The advantage in using Chenomx for these metabolites is that it accounts for quantification error by fitting experimental spectra of pure compounds to all the resonant peaks for the metabolite [29]. The statistical analysis presented later was applied to the peak integrals for all the metabolites, except for the metabolites cited above, for which Chenomx values were used.

^1^H NMR spectroscopic signals were assigned to metabolites after reference to the literature [30,31] or online databases (HMDB) [32], and/or confirmation by 2D NMR experiments on a selected sample, including homonuclear ^1^H-^1^H correlation spectroscopy and ^1^H-^1^H total correlation spectroscopy.

#### Statistical analysis

All statistical analyses were performed using R project software [33]. Continuous distributed variables were displayed as median with interquartile range and were tested using Mann–Whitney non-parametric statistical tests. Categorical variables were tested using the χ^2^ test. The threshold statistical significance was set at a *P* < 0.05 and conducted with a two-sided alternative hypothesis.

Statistical analyses were conducted on 34 metabolites to assess their variation in relation to birth outcomes (for example, PB, IPB, SPB and FGR) and to maternal parameters (biochemical measures and dietary intake). A five-step analysis was conducted to select metabolites that were significantly associated with birth outcomes and associated with metabolic syndrome. To identify metabolites associated with birth outcomes, a non-parametric test (Mann–Whitney U–test), was used, because of the non-normal distribution of the metabolite relative concentrations. The effect of multiple testing was considered by calculating the false discovery rate (FDR; that is, the expected proportion of the tests misclassified as significant for any given *P* value cut-off) [34]. To test for a dose–response association between metabolite levels and birth outcomes, a trend test (χ^2^ test) for trend in proportions, was used to assess the frequency distribution of women with pregnancy outcomes according to the quartiles of the metabolites [35]. For the metabolites identified as ‘of interest’ by the above analyses, their association with birth outcomes was tested after adjusting for confounding factors using multivariate logistic regression models. Interquartile range odds ratios (IORs) with 95% confidence intervals (CIs) were calculated for PB, IPB, SPB and FGR by using interquartile range for standardisation. We used the change from the outer quartiles as a measure, because metabolite integrals/predictors are not always normally distributed. Using the difference in the outer quartiles as a measure (0.25 and 0.75 quantiles), the OR is called the interquartile range or half-sample OR. Potential confounders with an established or potential association with PB or FGR were included in the logistic regression models. Receiver operator characteristic (ROC) curves and 95% CIs based on candidate metabolites (significant in logistic regression) were calculated for cases versus healthy controls using the package pROC in R [36].

In order to assess whether the metabolite panel associated with birth outcomes is also associated with known metabolic syndrome traits (BMI, BP, blood glucose, insulin, lipids), Spearman’s correlation coefficients were calculated. Metabolites with significant association with birth outcomes in logistic regression models and significant correlation coefficients with metabolic syndrome traits were selected for the final analysis. A stratified analysis by maternal BMI before pregnancy and maternal insulin levels at the first prenatal visit, was performed using multivariate logistic regression models on log-transformed metabolite levels, correcting for potential confounders (as described above).

## Results

### Descriptive statistics of the study population

Our metabolomics study was designed as a case–control study nested within the Rhea cohort. Table 1 shows the demographic characteristics within each case group, the control group and their comparison. Mothers of cases and controls tended to be of similar age (median 30 and 31 years, respectively), and (with the exception of SGA) possessed no significant differences in parity or in proportion of smokers. However, less educated women were more likely to develop pregnancy outcomes such as PB (32.7%) and FGR (27.8%) compared with controls (13.5%). The observations with respect to BMI and maternal education were consistent with associations reported in the wider cohort [18]. Extreme maternal BMI before pregnancy (either underweight or obese) occurred more in PB cases. In particular, more obese women had IPB (24% versus 11% in controls). Maternal BMI was not associated with FGR because maternal height and weight were accounted for in the assessment of FGR.

**Table 1:** Characteristics of the study population with respect to pregnancy outcomes

			**SPB (n = 88)**	**IPB (n = 26)**	**FGR (n = 36)**	**SGA (n = 19)**	**Control (n = 275)**
**Categorical variables**	Maternal education	Low	31 (35.6%)***	6 (23.1%)	10 (27.8%)*	4 (21.1%)	37 (13.5%)
		Medium	37 (42.5%)	11 (42.3%)	10 (27.8%)	7 (36.8%)	139 (50.5%)
		High	19 (21.8%)	9 (34.6%)	16 (44.4%)	8 (42.1%)	99 (36.0%)
	Greek origin		82 (93.2%)	26 (100.0%)	35 (97.2%)	19 (100.0%)	258 (94.2%)
	Multiparity		58 (65.9%)	19 (73.1%)	20 (55.6%)	12 (63.2%)	187 (68.0%)
	Smoking during pregnancy		19 (22.9%)	6 (24.0%)	10 (27.8%)	8 (42.1%)*	55 (20.4%)
	Pre-pregnancy BMI	Underweight	6 (7.2%)***	1 (4.0%)	1 (2.8%)*	1 (5.3%)	6 (2.2%)
		Normal	49 (59.0%)	10 (40.0%)	27 (75.0%)	15 (78.9%)	181 (66.8%)
		Overweight	16 (19.3%)	8 (32.0%)	4 (11.1%)	2 (10.5%)	54 (19.9%)
		Obese	12 (14.5%)	6 (24.0%)	4 (11.1%)	1 (5.3%)	30 (11.1%)
	FGR		3 (4.0%)	3 (14.3%)	36 (100.0%)***	17 (94.4%)***	0 (0.0%)
**Continuous variables**	Maternal age, years		29.0 (26.0 to 33.0)	31.0 (27.2 to 36.0)	30.0 (27.0 to 33.2)	30.0 (28.0 to 33.0)	31.0 (27.0 to 34.5)
	Gestational age, weeks		35.5 (35.0 to 36.0)***	36.0 (35.5 to 36.0)***	39.0 (37.5 to 40.0)	38.0 (38.0 to 40.0)	39.0 (38.0 to 39.0)
	Birth weight, g		2715 (2430 to 2980)***	2800 (2570 to 2890)***	2610 (2482 to 2802)***	2550 (2182 to 2615)***	3250 (3010 to 3550)
	Cholesterol (n = 227)		215.0 (189.5 to 237.0)	222.0 (212.8 to 233.0)*	205.0 (163.0 to 225.0)	225.0 (198.0 to 234.0)	202.0 (178.5 to 231.0)
	Triglycerides (n = 227)		112.0 (86.5 to 134.5)	149.0 (104.0 to 159.2)	99.0 (89.0 to 119.0)	95.0 (89.0 to 131.0)	111.0 (85.5 to 138.0)
	Insulin (n = 369)		6.3 (2.3 to 14.8)	10.6 (5.0 to 17.5)*	8.3 (3.2 to 26.6)	5.1 (2.7 to 37.9)	5.5 (2.0 to 15.9)
	LDL-C (n = 227)		128.0 (101.5 to 138.5)	130.0 (121.2 to 135.8)	116.0 (90.0 to 142.0)	142.0 (112.0 to 149.0)	114.0 (98.5 to 142.0)
	HDL-C (n = 227)		61.0 (52.0 to 71.0)	70.5 (59.8 to 79.2)	59.0 (49.0 to 69.0)	63.0 (49.0 to 67.0)	60.0 (49.0 to 68.5)
	Systolic BP (n = 338)		107.7 (101.0 to 115.7)	110.7 (105.7 to 117.3)	105.0 (96.0 to 112.1)	99.7 (94.2 to 110.2)	106.3 (100.3 to 112.0)
	Diastolic BP (n = 338)		69.7 (64.3 to 76.0)	74.5 (69.6 to 79.3)	69.3 (61.3 to 77.0)	65.2 (58.2 to 77.3)	69.7 (63.7 to 76.0)

Values are presented as medians (interquartile range) for continuous variables or frequencies, n (%) for categorical variables.

*P < 0.05 and ***P < 0.001 where *P* values were calculated using the χ^2^ test (categorical variables) or Mann–Whitney test (continuous) between cases and controls.

Gestational age and type of PB were known for 438 women, whereas FGR data were available for 401 women. BMI, body mass index; BP, blood pressure; FGR, fetal growth restriction; HDL-C, high density lipoprotein cholesterol; IPB, induced preterm birth; LDL-C, low density lipoprotein cholesterol; SGA, small for gestational age; SPB, spontaneous preterm birth.

### Metabolomic analysis

**Figure 1:**
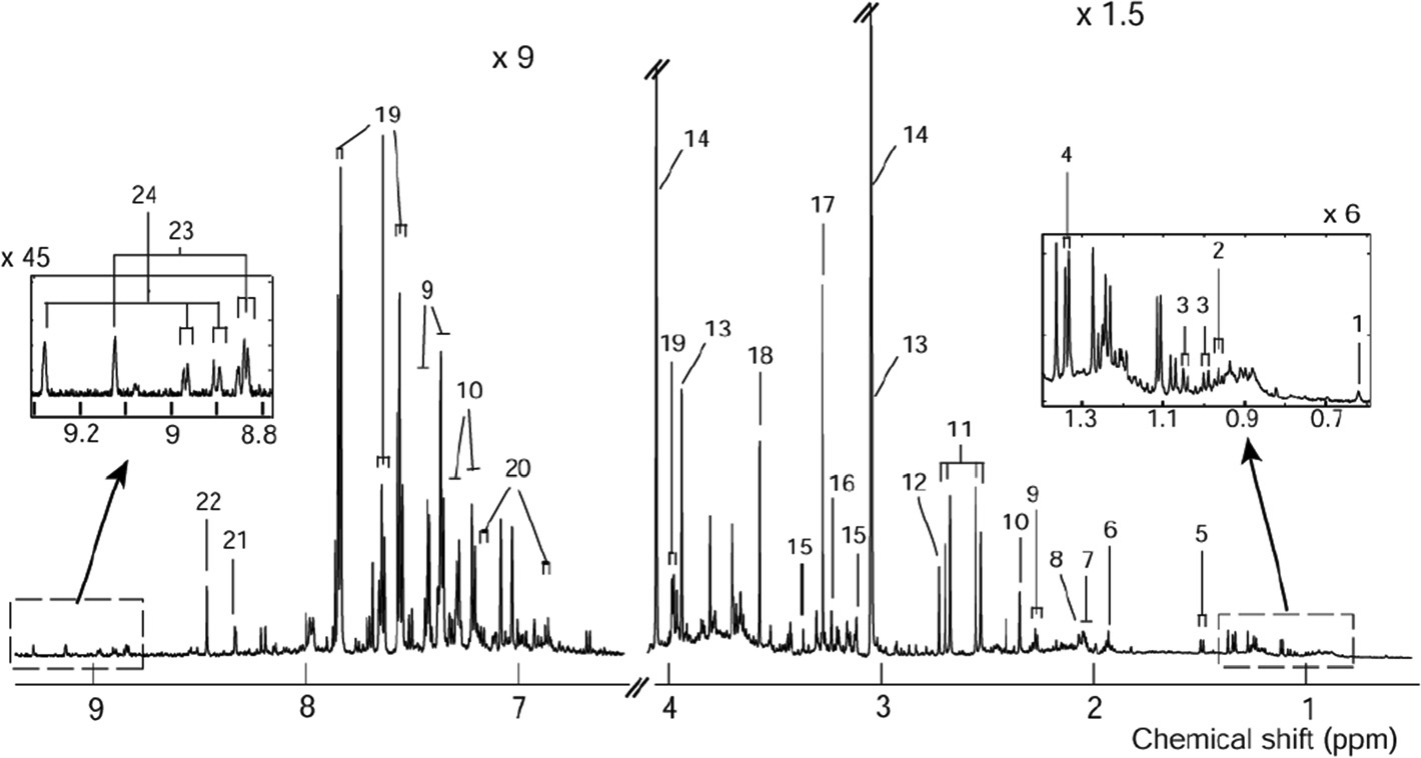
**Representative **^**1**^**H NMR spectrum (600 MHz) recorded for early pregnancy urine from a healthy pregnant woman.** Spectrum was recorded at 300 K. 1, Steroid conjugate; 2, leucine; 3, valine; 4, lactate; 5, alanine; 6, acetate; 7, *N-*acetyls of glycoprotein fragments; 8, *N-*acetyl neuraminic acid; 9, phenylacetylglutamine; 10, *p*-cresol sulphate; 11, citrate; 12, dimethylamine; 13, creatine; 14, creatinine; 15, proline betaine; 16, choline-containing moieties; 17, trimethylamine-*N-*oxide (TMAO); 18, glycine; 19, hippurate; 20, tyrosine; 21, *N-*methyl-2-pyridone-5-carboxamide (2Py); 22, formate; 23, *N-*methyl nicotinic acid (trigonelline); 24, 1-methylnicotinamide.

To obtain metabolic profiles, ^1^H NMR spectroscopy was applied to all the urine specimens from our study population (n = 464). From these spectra, 26 were excluded because of high dilution or high excretion of drug metabolites, leaving 438 available for modelling the metabolite profile with respect to birth outcomes. In total, 34 metabolites were identified in the urinary ^1^H NMR spectra (a representative assigned spectrum from a healthy pregnant woman is displayed in Figure 1). These included organic acids such as acetate, citrate and hippurate; aliphatic amines such as creatinine, DMA, trimethylamine (TMA) and trimethylamine-*N-*oxide (TMAO); amino acids such as alanine, leucine and tyrosine; and other metabolites such as *p*-cresol sulphate and niacin metabolites (*N-*methyl-2-pyridone-5-carboxamide or 2-Py).

A systematic analysis was performed to detect associations between birth outcomes (PB, IPB, SPB, SGA and FGR) and metabolite abundance. Because a single molecular species may give rise to multiple resonances (peaks) in an NMR spectrum, we chose to select a single representative peak for each metabolite (based on sufficient intensity and absence of overlap with other signals) to provide the measurement, with most metabolites exhibiting well-resolved peaks analysed by spectral integration. Our strategy was to use two different univariate approaches for initial candidate selection, and to look for agreement between these to define a consensus set of metabolites. These candidate metabolites were then subject to multivariate regression analysis to control for major confounding in our study. The first selection approach tested for significant median differences in metabolite abundance between cases and controls for each outcome. For a full description of the results of this analysis and the integral regions used for all metabolites, see Additional file 1. Of an initial 34 metabolites, eight metabolites displayed significant median differences between FGR and controls (see Table 2). Five metabolites were significant for both SGA and FGR, and two metabolites were specifically associated with SGA, namely leucine and *N-*acetyl neuraminic acid.

**Table 2:** Urinary metabolites with significant median differences between birth outcome cases and controls

	**Selected resonance (*δ), ppm***	**All PB types (n = 114)**			**SPB (n = 88)**			**IPB (n = 26)**			**FGR (n = 36)**			**SGA (n = 19)**		
		***P *** **value**	**q value**	**% diff**	***P *** **value**	**q value**	**% diff**	***P *** **value**	**q value**	**% diff**	***P *** **value**	**q value**	**% diff**	***P *** **value**	**q value**	**% diff**
Tyrosine^a^	6.87 (d), 7.18 (d)	0.055	0.264	−15%	0.161	0.410	−12%	0.083	0.530	−20%	**0.008**	**0.024**	**−24%**	0.094	0.220	−25%
Steroid conjugate – 0.63 (s)	0.63 (s)	**0.039**	**0.252**	**17%**	**0.045**	**0.218**	**19%**	0.391	0.808	11%	0.129	0.096	15%	0.392	0.486	8%
Leucine	0.96 (t)	0.364	0.578	2%	0.227	0.463	2%	0.778	0.893	−2%	0.443	0.226	1%	**0.026**	**0.093**	**11%**
Lactate	1.33 (d)	0.342	0.562	−5%	0.259	0.482	−5%	0.988	0.914	−4%	**0.025**	**0.051**	**−19%**	**0.009**	**0.081**	**−19%**
Alanine	1.48 (d)	0.627	0.702	−1%	0.426	0.602	−2%	0.653	0.875	3%	**0.031**	**0.055**	**−10%**	**0.027**	**0.094**	**−9%**
Lysine	1.73 (m)	0.056	0.264	1%	**0.016**	**0.214**	**2%**	0.762	0.891	−2%	0.349	0.197	−4%	0.512	0.553	0%
Acetate	1.92 (s)	0.423	0.613	−2%	0.885	0.758	0%	0.106	0.564	−7%	**0.003**	**0.018**	**−9%**	**0.017**	**0.081**	**−11%**
*N-*acetyl glycoprotein fragments	2.04 (s)	0.390	0.594	3%	0.783	0.735	0%	**0.009**	**0.231**	**9%**	0.358	0.200	−4%	0.913	0.688	−1%
*N-*acetyl neuraminic acid	2.06 (s)	0.806	0.752	0%	0.701	0.713	0%	0.173	0.650	7%	0.525	0.248	1%	**0.015**	**0.081**	**10%**
Citrate	2.55 (d)	0.818	0.754	−2%	0.882	0.757	−1%	0.395	0.809	−5%	**0.045**	**0.067**	**−9%**	0.119	0.250	−9%
Trimethylamine	2.87 (s)	0.094	0.277	−2%	0.218	0.457	−1%	0.133	0.593	−7%	**0.002**	**0.018**	**−18%**	**0.013**	**0.081**	**−17%**
Trimethylamine-*N-*oxide	3.27 (s)	0.067	0.270	−3%	**0.032**	**0.217**	**−3%**	0.976	0.913	6%	0.184	0.126	−7%	0.084	0.206	−9%
Glycine	3.57 (d)	0.103	0.279	−5%	**0.049**	**0.218**	**−10%**	0.920	0.908	8%	**0.019**	**0.044**	**−14%**	**0.008**	**0.081**	**−17%**
Phenylacetylglutamine	7.37 (d)	0.071	0.271	−9%	0.356	0.558	−3%	**0.015**	**0.231**	**−21%**	0.871	0.339	−1%	0.428	0.508	−12%
*N-*methyl-2-pyridone-5-carboxamide	8.33 (s)	0.065	0.269	8%	**0.049**	**0.218**	**8%**	0.676	0.879	−3%	0.764	0.311	−7%	0.545	0.568	−10%
Formate	8.46 (s)	**0.004**	**0.105**	**−11%**	**0.009**	**0.214**	**−12%**	0.115	0.574	−8%	**0.007**	**0.024**	**−16%**	0.410	0.498	−2%

% diff – percentage difference in group median.

Metabolite differences with *P* < 0.05 (Mann–Whitney test) are in bold type.

q Values indicate false discovery rate (likelihood of a false positive finding).

^a^Metabolite levels were determined by peak integration except for tyrosine, for which the Chenomx NMR suite was used.

FGR, fetal growth restriction; IPB, induced preterm birth; SGA, small for gestational age; SPB, spontaneous preterm birth.

Letters in brackets in the selected resonance column represent the signal multiplicity with (s) for singlets, (d) for doublets, (m) for multiplet and (t) for triplets.

The analysis of PB outcomes was conducted both on the combined clinical subtypes (PB) and separately on each subtype (SPB and IPB). Formate and an unassigned singlet resonance at 0.63 parts per million (ppm) probably derived from a steroid moiety, displayed significant (*P* < 0.05) median differences between PB and control cases (Mann–Whitney test, Table 2). Formate, *N-*methyl-2-pyridone-5-carboxamide (2-Py), glycine, TMAO, lysine and the singlet at 0.63 ppm significantly varied between SPB and control groups. The IPB group exhibited specifically higher levels of *N-*acetyl glycoproteins and lower levels of phenylacetylglutamine compared with controls. Using FDR analysis, we also estimated the likelihood of each difference between the groups being a false positive association (q values in Table 2); for significant metabolites in this analysis, these were observed to be up to 25% for IPB and SPB, up to 10% for SGA, and up to 7% for FGR.

For the metabolites with significant differences in pairwise tests, we next examined the trend in the proportion of women with each type of pregnancy outcome with increasing metabolite levels (dataset split in quartiles). Out of the eight candidate metabolites for FGR and the two metabolites applying to both PB and IPB, all showed a trend in frequency of birth outcomes across quartiles, therefore showing a dose–response relationship between levels of candidate metabolites and the outcome incidence. However, only three of the six candidate metabolites for SPB, namely formate, lysine and the singlet at 0.63 ppm, showed a significant trend (Table 3).

**Table 3:** Dose–response relationships between levels of selected metabolites and frequency of birth outcomes

**Outcome**	**Metabolite**	**Q1**	**Q2**	**Q3**	**Q4**	***P *** **value for trend**
All PB types (n = 114)	Steroid conjugate: 0.63 (s)	**21%**	**22%**	**25%**	**32%**	0.01*
	Formate	**32%**	**25%**	**23%**	**20%**	0.02*
SPB (n = 88)	Steroid conjugate: 0.63 (s)	**20%**	**22%**	**24%**	**34%**	0.01*
	Lysine	**14%**	**30%**	**26%**	**31%**	0.03*
	Trimethylamine-*N-*oxide	26%	28%	28%	17%	0.10
	Glycine	26%	34%	20%	19%	0.07
	*N-*methyl-2-pyridone-5-carboxamide	**19%**	**19%**	**30%**	**32%**	0.03*
	Formate	**32%**	**25%**	**24%**	**19%**	**0.02**
IPB (n = 26)	*N-*acetyl glycoprotein fragments	**8%**	**23%**	**19%**	**50%**	0.01**
	Phenylacetylglutamine	**42%**	**27%**	**12%**	**19%**	0.03*
FGR (n = 36)	Tyrosine	**33%**	**36%**	**17%**	**14%**	0.03*
	Lactate	**33%**	**33%**	**17%**	**17%**	0.05*
	Alanine	**39%**	**31%**	**11%**	**19%**	0.03*
	Acetate	**39%**	**31%**	**22%**	**8%**	0.004**
	Citrate	**33%**	**39%**	**14%**	**14%**	0.02*
	Trimethylamine	**50%**	**19%**	**17%**	**14%**	0.002**
	Glycine	**33%**	**39%**	**11%**	**17%**	0.03*
	Formate	**39%**	**28%**	**19%**	**14%**	0.02*
SGA (n = 19)	Leucine	16%	16%	26%	42%	0.06
	Lactate	**37%**	**37%**	**16%**	**11%**	0.05*
	Alanine	**42%**	**32%**	**16%**	**11%**	0.03*
	Acetate	**42%**	**21%**	**32%**	**5%**	0.05*
	*N-*acetyl neuraminic acid	**21%**	**11%**	**16%**	**53%**	0.04*
	Trimethylamine	47%	16%	21%	16%	0.07
	Glycine	**42%**	**37%**	**11%**	**11%**	0.02*

Percentage of each birth outcome frequency across pregnant women stratified by metabolite levels (into quartiles: Q1, Q2, Q3, and Q4).

*P < 0.05 and **P < 0.01 for a significant trend in outcome (Cochrane-Armitage trend test, highlighted in bold).

FGR, fetal growth restriction; IPB, induced preterm birth; SGA, small for gestational age; SPB, spontaneous preterm birth.

Finally, risk estimates of pregnancy outcomes were then computed using candidate metabolites as predictors in a logistic regression model, allowing adjustment for confounding factors such as maternal education, maternal age, parity and smoking habits (Table 4). The IORs between the outer quartiles (0.25 and 0.75 quantiles) of the candidate metabolite level was used to determine a significant association. Models for FGR indicated that high levels of tyrosine, acetate, trimethylamine and formate were significantly associated with a decreased incidence of FGR (IORs between 0.27 and 0.14). High levels of *N-*acetyl glycoproteins were associated with a dramatically increased risk of IPB (IOR = 5.84, 95% CI 1.44 to 39.5). High lysine and low formate levels were significantly associated with a higher risk of SPB.

**Table 4:** Logistic regression models predicting pregnancy outcomes from metabolite levels

**Outcomes**	**Metabolite**	**IQR**	**95% CI**		***P *** **value**
			**Min**	**Max**	
**All PB types (n = 114)**	Steroid conjugate: 0.63 (s)	1.90	0.99	3.69	0.054
	Formate	**0.51**	**0.26**	**0.99**	**0.047**
**SPB (n = 88)**	Steroid conjugate: 0.63 (s)	1.99	0.94	4.32	0.076
	Lysine	**2.79**	**1.20**	**6.98**	**0.021**
	*N-*methyl-2-pyridone-5-carboxamide	2.05	0.96	4.51	0.066
	Formate	**0.42**	**0.19**	**0.94**	**0.037**
**IPB (n = 26)**	*N-*acetyl glycoprotein fragments	**5.84**	**1.44**	**39.50**	**0.028**
	Phenylacetylglutamine	0.37	0.09	1.28	0.131
**FGR (n = 36)**	Tyrosine	**0.27**	**0.08**	**0.81**	**0.025**
	Lactate	0.37	0.12	1.04	0.069
	Alanine	0.38	0.13	1.02	0.064
	Acetate	**0.18**	**0.04**	**0.60**	**0.011**
	Citrate	0.33	0.09	0.99	0.058
	Trimethylamine	**0.14**	**0.04**	**0.40**	**0.001**
	Glycine	0.36	0.11	1.02	0.062
	Formate	**0.24**	**0.07**	**0.71**	**0.014**
**SGA (n = 19)**	Lactate	0.20	0.03	0.89	0.055
	Alanine	0.19	0.03	0.88	0.055
	Acetate	**0.12**	**0.01**	**0.70**	**0.050**
	*N-*acetyl neuraminic acid	2.23	0.64	9.10	0.225
	Glycine	0.19	0.03	0.88	0.052

Interquartile odds ratios (IQR, first versus fourth) with 95% confidence interval (CIs) are presented for the incident risk for pregnancy outcomes according to candidate metabolite relative concentrations.

Statistical analysis (z-score) of the beta values (log odds) indicated if the metabolite was significantly contributing to the model (highlighted in bold).

Models were adjusted for maternal education, maternal age, parity and smoking. FGR, fetal growth restriction; IPB, induced preterm birth; SGA, small for gestational age; SPB, spontaneous preterm birth.

**Figure 2:**
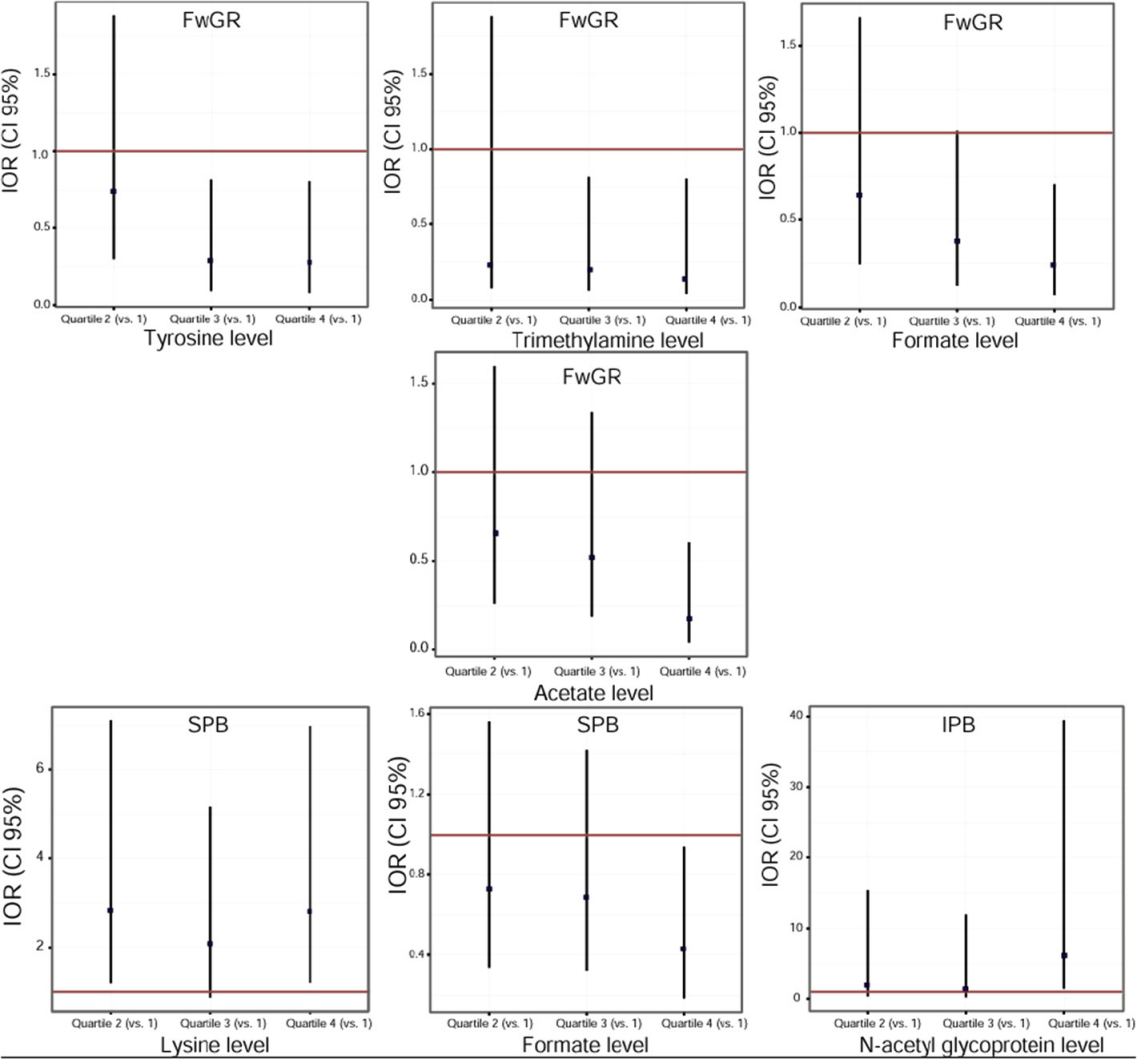
**Interquartile odds ratios (IORs) for pregnancy outcomes according relative concentrations of discriminatory urinary metabolites.** Logistic regression models were adjusted for maternal education, maternal age, parity and smoking. CI, 95% confidence interval.

IORs between all quartiles for metabolites significantly discriminating pregnancy outcomes are presented in Figure 2. Some metabolites, such as 2-Py in SPB cases, were associated with a linear increase in outcome incidence, whereas other metabolites, such as *N-*acetyl glycoproteins in IPB cases, were associated with a steep increase in outcome incidence only at high level. ROC analysis was also performed on the metabolites that were significantly associated in adjusted logistic regression models, in order to provide an alternative test of the ability of these molecules to predict birth outcomes in the study population. Modest but statistically significant area under the curve (AUC) values were obtained for all metabolites (AUCs for SPB: 58.8% to 59.4%; IPB: AUC 66%; FGR: 63.7% to 66.3%; see Additional file 2).

### Urinary metabolites characterising pregnancy outcomes and adverse metabolic status

**Figure 3:**
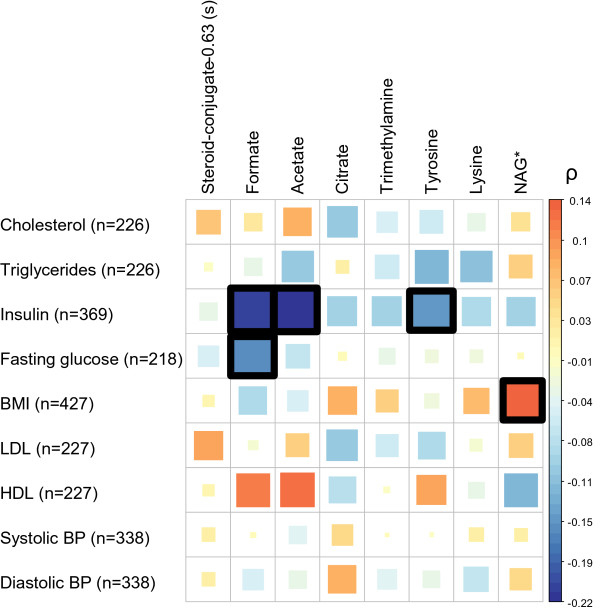
**Spearman’s correlation heatmap between metabolic syndrome components and urinary metabolites significantly associated with pregnancy outcomes.** Size and colour of each square indicates the magnitude of the correlation coefficient. Black outlined squares indicate *P* < 0.05 *NAG - N-acetyl-glycoprotein.

**Figure 4:**
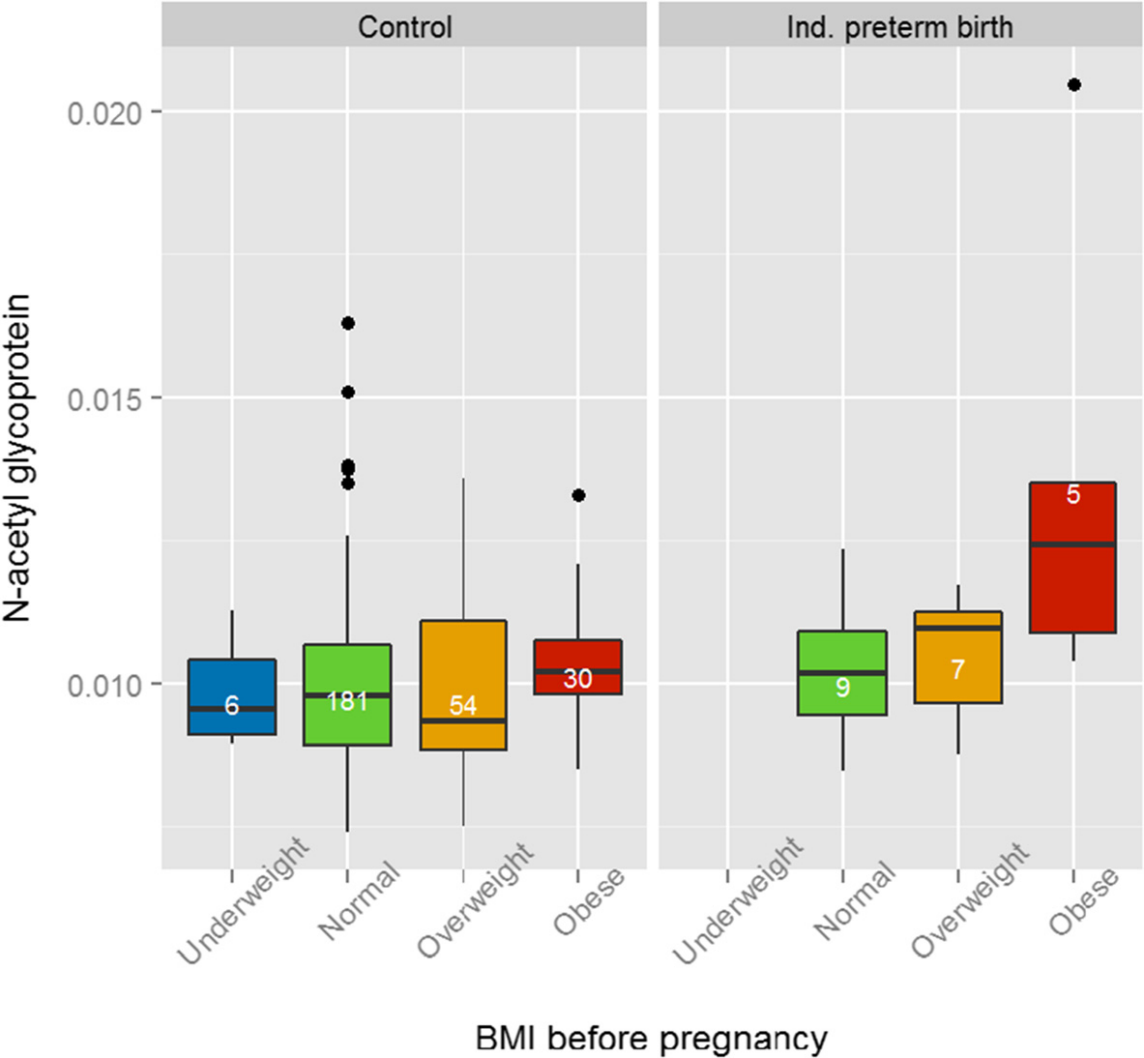
**Distribution of urinary *N-*** **acetyl glycoprotein resonance intensity in induced preterm birth (IPB) cases and controls stratified by maternal body mass index (BMI).** Box plots represent median and range of metabolite concentration with numbers in white corresponding to individual counts per categories.

According to a previous analysis [18], the presence of metabolic syndrome in early pregnancy is related to increased risk of PB and FGR within the Rhea cohort participants. Metabolic syndrome is a cluster of metabolic abnormalities related to increased risk of cardiovascular diseases and diabetes [37]. We hypothesised that the candidate metabolites associated with pregnancy outcomes might reflect aspects of metabolic syndrome, and that clinical parameters associated with metabolic syndrome would correlate with levels of the urinary metabolites (Figure 3). Insulin was the parameter with the most significant correlations with urinary metabolites, showing significant negative correlations with acetate, formate and tyrosine levels (Spearman ρ = −0.22, ρ = −0.21, and ρ = −0.15 respectively, *P* < 0.05). Increased BMI was associated with elevated levels of *N-*acetyl glycoprotein fragments in the urine (ρ = 0.14). BP was poorly correlated with urinary metabolites. These findings suggest that some of the variation in the urinary metabolites associated withbirth outcomes could be related to underlying maternal metabolic disease such as obesity and insulin resistance. Stratified analysis by maternal BMI as the two categories of ‘underweight and normal’ (<25) versus ‘overweight and obese’ (>25), confirmed that *N-*acetyl glycoprotein and IPB are significantly associated in overweight and obese women only (*P* = 0.008 in the overweight and obese group versus *P* = 0.40 in the underweight and normal group). Figure 4 illustrates that *N-*acetyl glycoprotein levels were particularly high in IPB women with high BMI before pregnancy. A stratified analysis was also performed for insulin levels (low levels ≤6 mU/mL versus high levels >6 mU/mL). Tyrosine, acetate and formate associations with FGR were not significant in the high insulin group.

## Discussion

Although over 90% of fetal growth occurs in the second half of gestation, maternal metabolism in the first trimester undergoes extensive changes in lipid storage, nitrogen species excretion and other metabolic pathways in order to facilitate fetal development [38]. Thus, early maternal metabolic abnormalities could indicate, or even cause, abnormal implantation, fetal growth impairment or other adverse birth outcomes, before clinical symptoms appear. Using a ^1^H NMR-based metabolic profiling approach, we found early (end of first trimester) differences in urinary metabolic phenotypes in the pregnant women in the Rhea cohort study in whom PB and FGR subsequently occurred. These potentially predictive metabolic signatures of birth outcomes were correlated with aspects of metabolic syndrome. Furthermore, we observed a distinction between the metabolic signature of ‘medically indicated’/induced and ‘non-indicated’/spontaneous PB, suggesting a range of aetiological metabolic factors contributing to PB.

Despite the relative small number of induced preterm pregnancies (n = 28), a significant increase in *N-*acetyl glycoprotein fragments was observed in these women. The *N-*acetyl proton resonances arising at δ 2.04 ppm are frequently associated with inflammation-induced acute phase proteins such as alpha-1 glycoprotein when reported in serum [39], but the urinary source is less certain. One candidate is uromodulin, also called the Tamm-Horsfall glycoprotein, which is the most abundant protein found in urine [40]. The *N-*acetyl glycoprotein resonance was positively correlated in this study with BMI. In the Rhea cohort, pregnant women with metabolic syndrome (and in particular the obesity component) had a high risk of encountering IPB (RR = 5.13, 95% Cl 1.97 to 13.38). The mechanisms relating *N-*acetyl glycoproteins to obesity and IPB, remain unclear; however, it is widely reported that high levels of adipose tissue can lead to systemic inflammation through release of cytokines such as interleukin-6, which could lead to an acute phase response [41]. Higher *N-*acetyl glycoprotein levels in NMR spectra of women with PB were also found in a study profiling maternal serum and cord blood at birth [42]. Spontaneous PB was specifically associated with higher urinary lysine, an essential amino acid that is limiting for growth, and is elevated in the plasma of premature infants [43]. The steroid conjugate at 0.63 ppm, possibly arising from a soluble metabolite of pregnanediol, was also increased in SPB cases by 19%. This signal has also been identified in previous studies that detected it in the urine of second trimester pregnant women with subsequent fetal malformation and trisomy 21 [20,44]. In our study, this steroid was also positively correlated with cholesterol and LDL-C, known sources for progesterone synthesis by the placenta.

With the exception of formate, a different metabolic profile (decreased urinary acetate, citrate, formate, glycine, tyrosine and trimethylamine) was associated specifically with poorer fetal growth. FGR remains difficult to assign owing to healthy biological variability in human size, hence in this study we used a definition based on customised birth-weight percentiles designed to better differentiate between infants who are small because of restricted growth and infants who are small but have reached their likely individual growth potential (see Methods) [18,26]. A similar pattern of associations was observed for this parameter as for the more conventional classification of SGA; however, FGR resulted in more statistically significant associations because of larger sample size*.* The FGR metabolite profile was broadly inversely associated with plasma insulin and positively correlated with HDL-C levels. Of these metabolites, formate, tyrosine and trimethylamine were all found to be significantly positively correlated with each other, suggesting a common source of variation (ρ_(formate-tyrosine)_ = 0.38, ρ_(formate-trimethylamine)_ = 0.21 and ρ_(tyrosine-trimethylamine)_ = 0.26). Elevations of several of these metabolites in blood have been previously associated with risk of insulin resistance [45,46]; however, the biological significance of low urinary levels of these molecules is less clear. Low urinary formate has been previously associated with increased hypertension in a large multinational study [47] and interestingly, hypertension in the first trimester of pregnancy was the most significant risk factor for PB and FGR in the Rhea cohort [18]. However, the association between formate and BP observed was not statistically significant in our study cohort. Several of the metabolites in the FGR signature (acetate, formate, tyrosine, trimethylamine) are known to be consumed or produced in significant quantities by gut microbes [48-51], hence the association might reflect a specific gut microbial distribution or a dietary pattern that selects for such a distribution. A recent study reported dramatic change in gut microbial composition throughout pregnancy causing increased insulin resistance and greater adiposity; although normally associated with disease this may be of benefit during pregnancy [52]. This indicates that the composition of gut microbiota in pregnant women could influence their metabolic homeostasis and their pregnancy outcomes. Daily intake of 5 mg of supplemental folic acid in the whole Rhea population (n = 1,279) was associated with a 66% decrease in the risk of delivering an SGA neonate (RR = 0.34; 95% CI 0.16 to 0.73) [53]. However, formate levels were not correlated with supplementary folate intake in our study population (ρ = −0.05 and *P* = 0.23).

Despite our study not being directly comparable with previous metabolomics studies investigating birth outcomes, owing to differences in the analytical platform used and the biofluids studied (often cord blood serum or amniotic fluid), and because our samples were taken at the end of the first trimester (whereas most previous metabolomics studies have examined late pregnancy samples), some similarities with other investigations were observed. In addition to the instances cited above, a previous study in women with subsequent FGR also reported decreased levels of urinary trimethylamine, tyrosine and glycine [44]. However, many more metabolomic studies have focused on events during pregnancy such as pre-eclampsia rather than birth outcomes; in our work, we excluded women with pre-eclampsia, making comparison with these inappropriate.

Our study has a number of other important limitations. Firstly, our study was not specifically designed to examine FGR, and only a limited number of these cases were available within our dataset. Secondly, although our study is unique in defining associations between metabolism during early pregnancy and birth outcomes, it is not possible at this stage to distinguish between pregnancy-induced effects and underlying metabolic risk factors. However, this does not negate the potential value of urinary metabolites in general as biomarkers of risk of negative birth outcomes, and our exploratory study has generated several hypotheses for future investigation. It is also possible that our observations reflect aetiological factors specific to the Rhea cohort, which experience an abnormally high rate of PB, and are not generalisable to the broader European population. Specimens from an independent cohort would be needed to validate our findings, and several efforts to complete comparable studies in large birth cohorts are currently underway, such as the Human Early-Life Exposome (HELIX) project [54]. This consortium aims to implement novel exposure assessment and biomarker methods (including metabolomics) to characterise the total exposure from conception to multiple environmental factors (the exposome [55-57]) and associate these with child health outcomes. Applied as an untargeted approach, metabolomics captures information about endogenous metabolism and exogenous exposures simultaneously, making it in principle an ideal tool for exploring the exposome.

## Conclusion

Urinary acetate, tyrosine, formate, trimethylamine, lysine and glycoprotein measured at the end of the first trimester are associated with increased risk of negative birth outcomes in the Rhea cohort. We believe our study to be a confirmation of the potential of metabolomics to reveal novel links between metabolite exposure and birth outcomes, and evidence in support of the inclusion of such approaches in studies that attempt to link the exposome to neonatal health.

## Abbreviations

2-Py: *N-*methyl-2-pyridone-5-carboxamide; FGR: Fetal weight growth restricted; IPB: Induced preterm birth; NMR: Nuclear magnetic resonance; PB: Preterm birth; SPB: Spontaneous preterm birth. BMI body mass index; BP: blood pressure; HDL-C: high density lipoprotein cholesterol; LDL-C: low density lipoprotein cholesterol; SGA: small for gestational age; PB: spontaneous preterm birth.

## Competing interests

The authors declare that they have no competing interests.

## Authors’ contributions

LM carried out the experiments, analysed the data, and drafted the manuscript. MK & LC designed the Rhea cohort study. HK, MK, EF & LC conceived this metabonomic study, and participated in its design and coordination. EF compiled the epidemiological and clinical data. TA supervised the laboratory work and drafted the manuscript. EH helped with data interpretation and helped to draft the manuscript. MC contributed to the NMR spectral assignment and drafted the manuscript. HK contributed to the statistical analysis and drafted the manuscript. MBT provided her expertise in reproductive epidemiology and statistical analysis. All authors read and approved the final manuscript.

## Supplementary Material

Additional file 1 Analysis of median differences (Mann-Whitney U test) for all metabolite integrals comparing negative birth outcome groups and controls. Click here for file

Additional file 2 Discrimination between birth outcome cases and control group receiver operating characteristic (ROC) curves for selected candidate metabolites. Click here for file
