# Supplementary material for: Urinary metabolic profiles in early pregnancy are associated with preterm birth and fetal growth restriction in the Rhea mother–child cohort study
Source: BMC Med. 2014 Jul 11;12:110. doi: 10.1186/1741-7015-12-110 (PMC4094172; doi:10.1186/1741-7015-12-110)
Supplement: Additional file 1 — Analysis of median differences (Mann-Whitney U test) for all metabolite integrals comparing negative birth outcome groups and controls. [file 1741-7015-12-110-S1.docx]

|  | Integral region δ (ppm) | | All PB types  (n=114) | | SPB  (n=88) | | IPB  (n=26) | | FWGR  (n=36) | | SGA  (n=19) | |
| --- | --- | --- | --- | --- | --- | --- | --- | --- | --- | --- | --- | --- |
| Metabolite | **δ1** | **δ2** | **p-value** | **q-value** | **p-value** | **q-value** | **p-value** | **q-value** | **p-value** | **q-value** | **p-value** | **q-value** |
| Steroid-conjugate | 0.599 | 0.645 | **0.039** | 0.252 | **0.045** | 0.218 | 0.391 | 0.808 | 0.129 | 0.096 | 0.392 | 0.486 |
| Unknown 0.75 (d) | 0.736 | 0.767 | 0.160 | 0.375 | 0.111 | 0.349 | 0.900 | 0.906 | 0.060 | 0.075 | 0.366 | 0.469 |
| Leucine | 0.95 | 0.98 | 0.364 | 0.578 | 0.227 | 0.463 | 0.778 | 0.893 | 0.443 | 0.226 | **0.026** | 0.093 |
| Valine | 0.98 | 1.01 | 0.672 | 0.716 | 0.282 | 0.500 | 0.245 | 0.724 | 0.539 | 0.252 | 0.279 | 0.403 |
| Unknown 1.08 (d) | 1.063 | 1.09 | 0.892 | 0.770 | 0.971 | 0.775 | 0.805 | 0.896 | 0.470 | 0.234 | 0.633 | 0.605 |
| 4-deoxyerythronic acid | 1.097 | 1.12 | 0.127 | 0.322 | 0.266 | 0.485 | 0.165 | 0.640 | 0.749 | 0.306 | 0.686 | 0.624 |
| Unknown 1.15 (d) | 1.132 | 1.16 | 0.577 | 0.684 | 0.606 | 0.682 | 0.778 | 0.893 | 0.097 | 0.088 | 0.172 | 0.294 |
| 3-hydroxyisovalerate | 1.264 | 1.3 | 0.969 | 0.784 | 0.639 | 0.693 | 0.393 | 0.808 | 0.681 | 0.287 | 0.704 | 0.630 |
| Lactate | 1.314 | 1.37 | 0.342 | 0.562 | 0.259 | 0.482 | 0.988 | 0.914 | **0.025** | 0.051 | **0.009** | 0.081 |
| 3-amino-isovalerate | 1.356 | 1.37 | 0.103 | 0.279 | 0.173 | 0.422 | 0.259 | 0.736 | 0.116 | 0.092 | 0.172 | 0.294 |
| Alanine | 1.472 | 1.51 | 0.627 | 0.702 | 0.426 | 0.602 | 0.653 | 0.875 | **0.031** | 0.055 | **0.027** | 0.094 |
| Lysine | 1.701 | 1.76 | 0.056 | 0.264 | **0.016** | 0.214 | 0.762 | 0.891 | 0.349 | 0.197 | 0.512 | 0.553 |
| Acetate | 1.915 | 1.94 | 0.423 | 0.613 | 0.885 | 0.758 | 0.106 | 0.564 | **0.003** | **0.018** | **0.017** | 0.081 |
| N-acetyl glycoprotein fragments | 2.016 | 2.05 | 0.390 | 0.594 | 0.783 | 0.735 | **0.009** | 0.231 | 0.358 | 0.200 | 0.913 | 0.688 |
| N-acetyl neuraminic acid | 2.054 | 2.08 | 0.806 | 0.752 | 0.701 | 0.713 | 0.173 | 0.650 | 0.525 | 0.248 | **0.015** | 0.081 |
| Acetone | 2.229 | 2.25 | 0.608 | 0.695 | 0.751 | 0.727 | 0.563 | 0.858 | 0.117 | 0.092 | 0.369 | 0.471 |
| *p*-cresol sulfate | 2.357 | 2.34 | 0.510 | 0.657 | 0.986 | 0.777 | 0.125 | 0.585 | 0.977 | 0.366 | 0.738 | 0.640 |
| Citrate | 2.516 | 2.59 | 0.818 | 0.754 | 0.882 | 0.757 | 0.395 | 0.809 | **0.045** | 0.067 | 0.119 | 0.250 |
| Trimethylamine | 2.86 | 2.88 | 0.094 | 0.277 | 0.218 | 0.457 | 0.133 | 0.593 | **0.002** | **0.018** | **0.013** | 0.081 |
| Choline | 3.224 | 3.24 | 0.210 | 0.441 | 0.145 | 0.394 | 0.969 | 0.912 | 0.597 | 0.265 | 0.843 | 0.671 |
| TMAO | 3.261 | 3.29 | 0.067 | 0.270 | **0.032** | 0.217 | 0.976 | 0.913 | 0.184 | 0.126 | 0.084 | 0.206 |
| Proline betaine | 3.311 | 3.29 | 0.844 | 0.760 | 0.797 | 0.738 | 0.323 | 0.776 | 0.084 | 0.084 | 0.160 | 0.285 |
| Glycine | 3.581 | 3.56 | 0.103 | 0.279 | **0.049** | 0.218 | 0.920 | 0.908 | **0.019** | **0.044** | **0.008** | 0.081 |
| Phenylacetylglutamine | 7.342 | 7.38 | 0.071 | 0.271 | 0.356 | 0.558 | **0.015** | 0.231 | 0.871 | 0.339 | 0.428 | 0.508 |
| Hippurate | 7.53 | 7.58 | 0.738 | 0.735 | 0.395 | 0.583 | 0.356 | 0.792 | 0.493 | 0.240 | 0.073 | 0.190 |
| Unknown 7.68(s) | 7.672 | 7.69 | 0.853 | 0.762 | 0.601 | 0.680 | 0.539 | 0.853 | 0.263 | 0.164 | 0.802 | 0.659 |
| 2Py | 8.311 | 8.35 | 0.065 | 0.269 | **0.049** | 0.218 | 0.676 | 0.879 | 0.764 | 0.311 | 0.545 | 0.568 |
| Formate | 8.45 | 8.48 | **0.004** | 0.105 | **0.009** | 0.214 | 0.115 | 0.574 | **0.007** | **0.024** | 0.410 | 0.498 |
| Trigonelline | 8.81 | 8.87 | 0.220 | 0.452 | 0.236 | 0.469 | 0.598 | 0.865 | 0.620 | 0.270 | 0.711 | 0.632 |

|  | Integral region δ (ppm) | All PB types  (n=114) | | SPB  (n=88) | | IPB  (n=26) | | FWGR  (n=36) | | SGA  (n=19) | |
| --- | --- | --- | --- | --- | --- | --- | --- | --- | --- | --- | --- |
| Metabolite |  | **p-value** | **q-value** | **p-value** | **q-value** | **p-value** | **q-value** | **p-value** | **q-value** | **p-value** | **q-value** |
| Creatine | Chenomx | 0.994 | 0.789 | 0.601 | 0.680 | 0.282 | 0.752 | 0.076 | 0.082 | 0.135 | 0.265 |
| Creatinine | Chenomx | 0.927 | 0.777 | 0.923 | 0.766 | 0.679 | 0.879 | 0.579 | 0.261 | 0.795 | 0.658 |
| Tyrosine | Chenomx | 0.055 | 0.264 | 0.161 | 0.410 | 0.083 | 0.530 | **0.008** | **0.024** | 0.094 | 0.220 |
| Dimethylamine | Chenomx | 0.335 | 0.557 | 0.450 | 0.614 | 0.443 | 0.826 | 0.113 | 0.091 | 0.625 | 0.602 |
| 1-methyl-nicotinamide | Chenomx | 0.868 | 0.765 | 0.783 | 0.735 | 0.874 | 0.904 | 0.052 | 0.071 | 0.542 | 0.567 |
