# Supplementary figures and images for: Urinary metabolic profiles in early pregnancy are associated with preterm birth and fetal growth restriction in the Rhea mother–child cohort study
Source: BMC Med. 2014 Jul 11;12:110. doi: 10.1186/1741-7015-12-110 (PMC4094172; doi:10.1186/1741-7015-12-110)

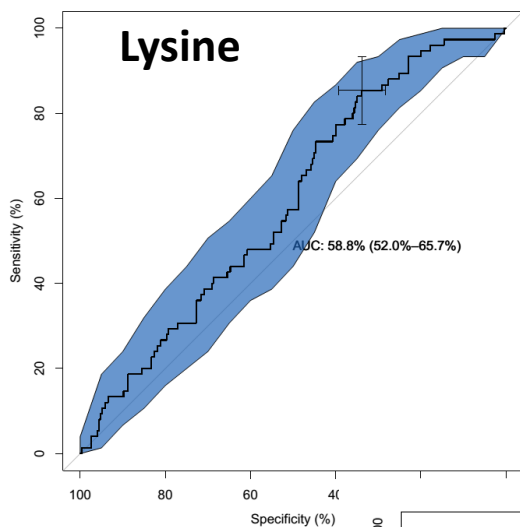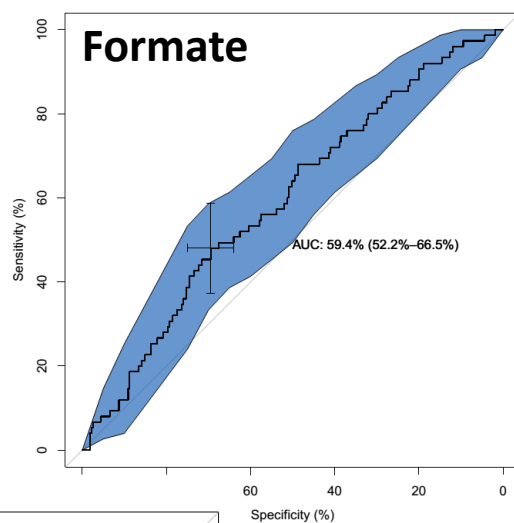

SPB

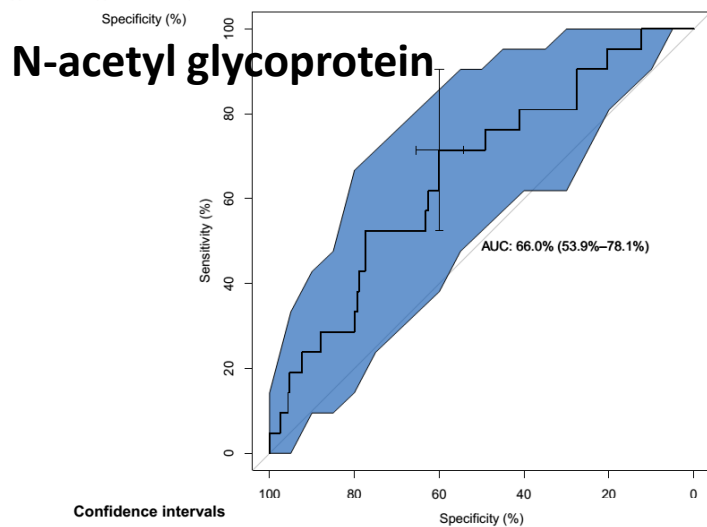

IPB

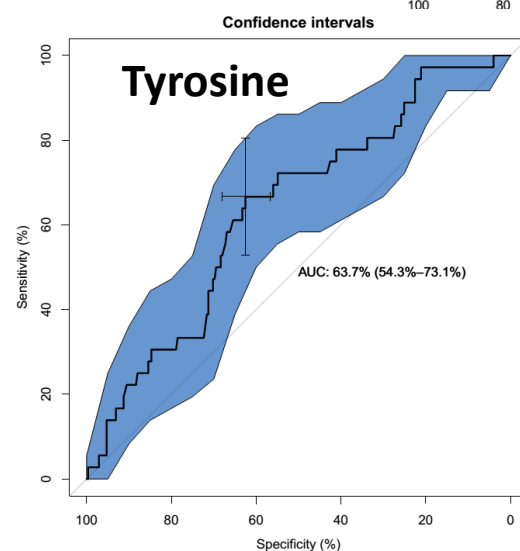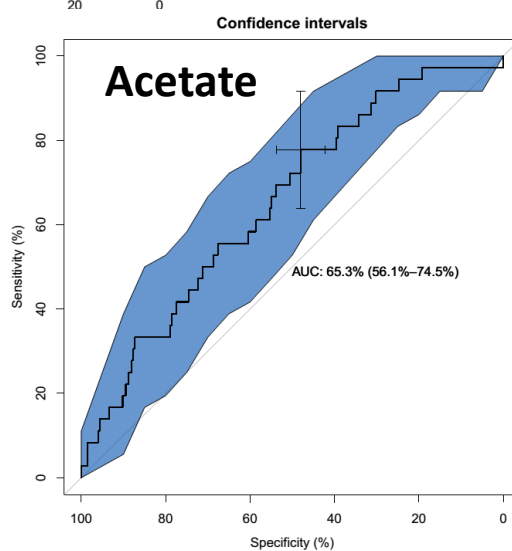

FGR

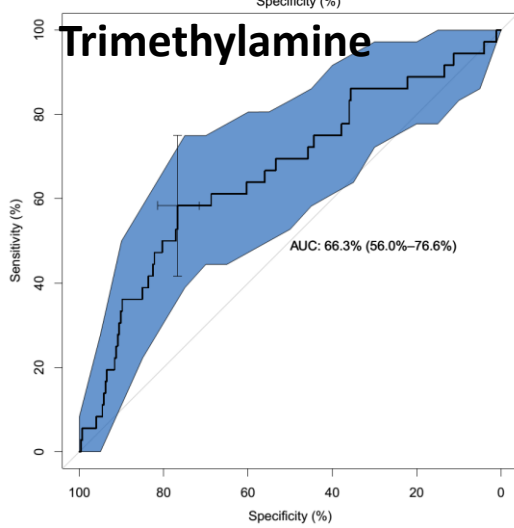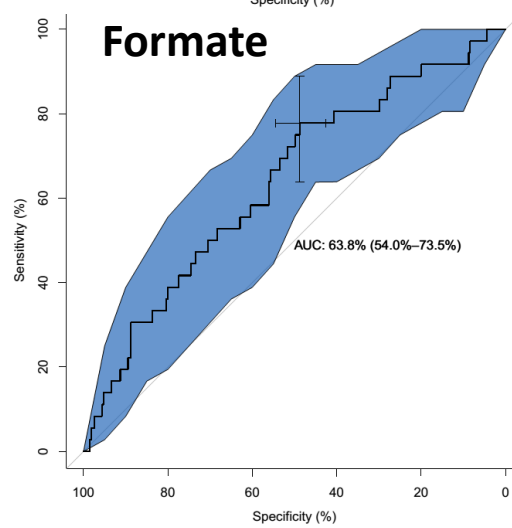

Supplement: Additional file 2 — Discrimination between birth outcome cases and control group receiver operating characteristic (ROC) curves for selected candidate metabolites. [file 1741-7015-12-110-S2.pdf]
